# Investigating a Participatory Intervention in Multidisciplinary Cancer Care Teams Using an Integrative Organizational Model: A Study Protocol

**DOI:** 10.3389/fpsyg.2022.798863

**Published:** 2022-05-03

**Authors:** Denis Chênevert, Tyler L. Brown, Marie-Pascale Pomey, Nadia Benomar, Philippe Colombat, Evelyne Fouquereau, Carmen G. Loiselle

**Affiliations:** ^1^Department of Human Resources, HEC Montreal, Montreal, QC, Canada; ^2^Healthcare Management Hub, HEC Montreal, Montreal, QC, Canada; ^3^Centre de Recherche du Centre Hospitalier de l’Université de Montréal (CRCHUM), Montreal, QC, Canada; ^4^Unité de Soutien SSA, Université de Sherbrooke, Campus de Longueuil, Longueuil, QC, Canada; ^5^Department of Oncology, Faculty of Medicine and Health Sciences, McGill University, Montreal, QC, Canada; ^6^Department of Management, Evaluation and Health Policy, School of Public Health, Université de Montréal, Montreal, QC, Canada; ^7^Department of Family Medicine and Emergency Medicine, Faculty of Medicine, Université de Montréal, Montreal, QC, Canada; ^8^Qualipsy EE 1901, Department of Psychology, Université de Tours, Tours, France; ^9^Ingram School of Nursing, Faculty of Medicine and Health Sciences, McGill University, Montreal, QC, Canada; ^10^Lady Davis Institute for Medical Research, Jewish General Hospital, Montreal, QC, Canada; ^11^Segal Cancer Centre, Jewish General Hospital, Montreal, QC, Canada

**Keywords:** cancer care teams, longitudinal design, managerial practice, mental health, mixed methods, participatory interventional approach, resilience, structural equation modeling

## Abstract

Multidisciplinary teams encounter many challenges that can lead to higher levels of distress and burnout. This trend is acutely prevalent among multidisciplinary cancer care teams who frequently contend with increased task complexity and numbers of patients. Resilience is emerging as a critical resource that may optimize team members’ psychological health and wellbeing, work efficiency, and organizational agility, while reducing burnout. Accordingly, the proposed study aims to implement and evaluate a promising participatory interventional approach that fosters team resilience. Specifically, the effects of the intervention on participating team members will be compared to a control group of non-participating team members. This intervention’s core components include skills training, patient-centered meetings, talking spaces, and an agile problem-solving approach. The proposed study also seeks to determine whether enhanced resilience improves team mental health status and organizational outcomes. A participatory interventional approach will be implemented and assessed at three-time intervals [i.e., pre-intervention deployment (*N* = 375), 12 months post-deployment (*N* = 236), and 24 months post-deployment (*N* = 146)] across five cancer care teams in three Quebec healthcare institutions. A mixed methods design will be used that includes observations, semi-structured interviews, focus groups, and self-report questionnaires. Direct observation will document team functioning and structural resources (e.g., meetings, conflict management, and leadership). Semi-structured interviews will explore participants’ experience with activities related to the participatory interventional approach, its perceived benefits and potential challenges. Focus groups will explore participants’ perceptions of their team’s resilience and the effectiveness of the intervention. Questionnaires will assess support, recognition, empowerment, organizational justice, individual resilience, psychological safety, work climate, team resilience, workplace burnout, engagement, quality of work life, wellbeing, and organizational citizenship behaviors, and sociodemographic variables. Moreover, objective measures including absenteeism and staff turnover will be obtained *via* human resource records. Structural equation modeling will be used to test the study’s hypotheses. The proposed protocol and related findings will provide stakeholders with quantitative and qualitative data concerning a participatory interventional approach to optimize team effectiveness. It will also identify critical factors implicated in favorable organizational outcomes in connection with multidisciplinary cancer care teams. Expected results and future directions are also presented herein.

## Background

Multidisciplinary cancer care teams are faced with the increasing complexity of treatment and service delivery organization (Berman et al., 2020), as well as a more significant number of individuals diagnosed (and living) with cancer. This increase in service demand, coupled with fewer organizational resources and increased psychological distress and loss of workplace meaning, can result in higher than average absenteeism, distress, and burnout (Ramirez et al., 1995; Shanafelt et al., 2015; Fillion et al., 2017). Indeed, healthcare professionals, including cancer care team members, often experience higher burnout and absenteeism rates relative to the general population (Ramirez et al., 1995; Chênevert et al., 2013; Shanafelt et al., 2015; Fillion et al., 2017). Moreover, amid the Covid-19 pandemic (and its associated containment measures), such challenges are compounded, thereby intensifying the urgency for understanding and managing the dynamics of team functioning in periods of crisis. Several avenues have been suggested to address these issues; however, none appear to have provided a comprehensive approach and solution (Chênevert et al., 2013).

**Figure 1:**
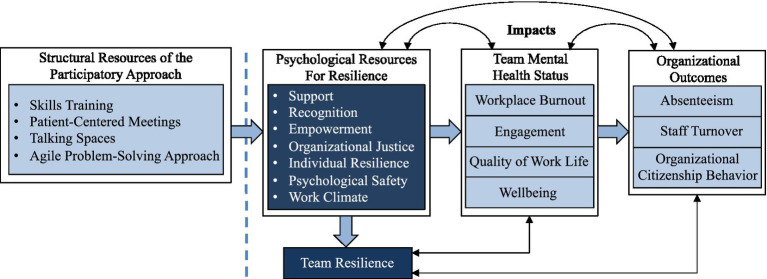
The proposed integrative organizational model.

Moreover, few studies have attempted to document the dynamics within and across cancer care teams concerning key constructs such as psychological safety, empowerment, and workplace quality of life. Furthermore, to our knowledge, few studies have identified associations among healthcare teams’ resilience, workplace wellbeing, and workplace behavior (Lejeune et al., 2019). Additionally, whereas Dubois et al. (2020) have proposed an analysis that considers similar variables and context, it does not appear to be informed by a comprehensive theoretical perspective (Chênevert et al., 2020) nor, as suggested by Lejeune et al. (2020), propose the testing of an integrative model and inter-relationships among variables. Hence, the present study aims to implement and evaluate an intervention for multidisciplinary cancer care teams intended to foster psychological resources of resilience, which in turn will foster team resilience, enhance mental health status, and augment organizational outcomes (Figure 1).

## Resilience as a Core Component of Effective Multidisciplinary Team Functioning

According to Horne and Orr (1998, p. 31), resilience refers to a “fundamental quality of individuals, groups, organizations, and systems as a whole to respond productively to significant change that disrupts the expected patterns of events without engaging in an extended period of regressive behavior.” Likewise, rather than signifying a fixed (or static) characteristic, resilience refers to a process that can be developed to adaptively react to and emerge from adverse situations (Vera et al., 2017). More specifically, team resilience conveys a psychological mechanism contingent on a range of factors and resources (e.g., individual, collective, and organizational) that modulate individual and team performance (Meneghel et al., 2016; Vera et al., 2017).

Developing team resilience requires the targeting of specific factors, including leadership style (Doucet et al., 2009; Vera et al., 2017), psychological safety (Hollnagel et al., 2019), relational climate (Meneghel et al., 2016), quality of interactions (Meneghel et al., 2016; Vera et al., 2017), and behavioral norms and meaning at work (Colombat, 2012; Gucciardi et al., 2018). Historically, leadership has been the responsibility of one high-status person exercising authority over a group of lower-status individuals (Colombat, 2012). However, this vertical conception of leadership no longer corresponds to the reality of multidisciplinary teams (Colombat, 2012), including cancer care teams. Indeed, the high degree of expertise possessed by team members, combined with an increased need for decisional autonomy, accentuates the need for shared leadership (Carson et al., 2007; Nissim et al., 2019). Psychological safety helps create an atmosphere where team members are encouraged to express their views and take interpersonal risks (e.g., speaking up in team meetings) unhindered by the threat of adverse reactions from higher status team members. In addition, psychological safety allows team members to become more creative, resilient, and collaborative, while also fostering their ability to solve complex problems as a high functioning unit that can draw on the collective knowledge of the group as a whole (O’Donovan and Mcauliffe, 2020). Relational climate (e.g., team climate and collaboration) has also been found to increase team resilience, which, in turn, enhances team performance (Meneghel et al., 2016). Quality of interaction (e.g., planning, coordination, collective reflection) also represents pathways to developing and sustaining resilience (Colombat, 2012). Furthermore, the quality of interaction among multidisciplinary team members possesses several vital factors, including members with complementary roles, regular meetings, and shared physical space (Smith et al., 2018). Additional resilience-based factors related to behavioral norms and meaning at work include respect, open communication, and a constructive problem-solving approach that supports team members (Smith et al., 2018). All of these factors are central to the participatory interventional approach informing this study’s intervention.

## The Participatory Interventional Approach

The participatory interventional approach used in the present study was first implemented in the French health system in the 1990s under the ethos that improved quality of health professionals’ life at work would result in the enhanced overall care of patients and their families (Lejeune et al., 2020). This approach, which increases work team resilience, is defined as a dynamic, person-centered, organizational model based on instilling meaning in work and allowing each individual to find their place in the team to prevent burnout through a higher work-related quality of life. The participatory interventional approach, developed and tested within multidisciplinary teams (Lejeune et al., 2021), is based on implementing several interdependent activities with the overarching goal of supporting and promoting team resilience.

The first step of this interventional approach includes mapping the processes to understand the actual work of teams rather than the prescribed work, thus making it possible to identify what is going well in the team rather than what is going poorly, which makes it possible to generate the anticipatory behaviors necessary for enhanced team resilience (Hollnagel et al., 2019). This step also allows us to analyze the teams’ governance system to understand the work climate and the initial environment before deploying the intervention. This first step will be part of the study’s approach (i.e., relying on focus groups and semi-structured interviews). The objective is to allow each selected cancer care team to contextualize the approach and undertake an initial (baseline) measurement phase (T0). We will also be able to identify each of the teams’ characteristics, which will allow us to qualify our analysis. Next, in collaboration with the organizations, the participatory approach’s different interdependent components (Table 1) will be deployed. Of note, intervention activities will be delivered based on the specifics of the team, as determined by the step outlined above. For instance, considering the next step, if team members have previously undergone mindfulness training, we will not repeat this training in the intervention.

**Table 1:** Interdependent components of the participatory interventional approach.

Components	Activities
Skills training	Train staff on individual resilience, mindfulness, and stress management Develop “in-house training” to share knowledge within the team Identify team relevant topics (patient care or technical) Identify team “expert” to share knowledge on relevant topic Organize training for each shift (1 h long; theory and discussion) Encourage team to develop a common ground and shared vernacular
Patient-centered meetings	Select complex patient cases (maximum: 25) Organize weekly meetings to discuss cases Select group leader (either physician or manager) Apply a hierarchically reversed group speaking order Enter all weekly meeting decisions into patient file
Talking spaces	Appoint external psychologist as facilitator Hold monthly volunteer meetings Review situations and personal reactions Work to make sense of experience Learn solutions to better cope/adjust
Agile problem-solving approach	Identify areas for improvement Select a specific problem Create a temporary multidisciplinary task focus Refine problem definition and objectives Conduct a root cause analysis Identify and implement solution(s) Disband temporary multidisciplinary task force Team assumes responsibility for monitoring solution(s)

### Skills Training: Individual Resilience, Mindfulness, and Stress Management

The foundation of the participatory interventional approach involves fostering individual resilience among team members *via* training on mindfulness, emotional intelligence, and stress management skills. This training aims to help team members internalize certain skills and attitudes associated with later intervention stages focused on improving organizational practices. Indeed, healthcare professionals who neglect their personal needs and experience burnout also reduce their overall cognitive availability level for their colleagues and patients (Nissim et al., 2019). Thus, although the research team will focus on individual resilience, it will situate this training in a top-down context that encourages associated institutions to formally support the approach (e.g., promotion *via* senior management or provision of training time). In-house training will also be emphasized to enhance staff engagement and awareness of various team knowledge sources.

### Patient-Centered Meetings

This activity will involve enhancing the quality of the multidisciplinary meetings already present or setting them up, if absent, so that two key dimensions are covered: a focus on patients and a focus on work dynamics and the quality of practices. To this end, existing meetings will be revised so that their duration, frequency, and objectives are better structured and professionals are actively involved (Bilodeau et al., 2015). Meetings that include the patient’s perspective must be aimed at the individual’s overall management (as opposed to only the disease condition; Loiselle and Brown, 2020). The participatory interventional approach assumes that these multidisciplinary meetings, based on the patient’s perspective, make room for the physical, psychological, social, occupational, and existential/religious-spiritual needs of patients and that all stakeholders can contribute to the discussion according to a participatory dynamic and a predetermined order of speaking (in reverse order of hierarchical position), to enable better decision-making based on consensus (Ceccaldi, 2015). These meetings must be complemented by meetings focused on the work dynamic and the quality of practices, which may take several forms depending on the context, e.g., peer group, practice analysis, or ethical reflection group.

Presently, multidisciplinary discussions are too often limited to treatment plans, prioritizing physicians’ input over other team members’ input. Drawing on everyone’s contribution, we encourage group reflection and learning and the implementation of an action plan common to the entire team. Ideally, effective incident learning leads to improvements in practice that result in greater safety and productivity (Lukic et al., 2012) as incident analysis seeks to reveal the contributing factors and underlying causes of the incident (Drupsteen and Guldenmund, 2014). To be effective, this process must be collaborative and participatory (Lukic et al., 2012; Macrae, 2015). However, for teams to develop resilience and adjust effectively to disruptions and unexpected events (Fairbanks et al., 2014; Hollnagel, 2014a,b; Sujan et al., 2015a,b), meetings must also be an environment where teams learn and value what goes well in daily clinical practice (Braithwaite et al., 2015; Wears et al., 2015).

### Talking Spaces

Team support can take different forms. In addition to informal hallway meetings, support among stakeholders is provided indirectly through multidisciplinary meetings, for which participation should be encouraged and promoted by the organization when possible (Ceccaldi, 2015). These meetings allow participants to link their practice to that of others, limiting the inconsistency of decision-making that can be a source of stress (Ceccaldi, 2015) and allowing participants to develop a common sense and pace of work (Pronost et al., 2008). Support can also be provided by organizations by offering group facilitation. To this end, a study conducted in pediatric oncology has established a link between talking spaces and the quality of patient care by increasing psychological resilience resources (Lejeune et al., 2019).

### The Agile Problem-Solving Approach

Implementing a project approach within teams means providing them with problem-solving tools based on continuous improvement. The emergence of projects within teams often stems from a variety of day-to-day problems. Currently, these issues are most often discussed during department meetings and are recurrent. Implementing an agile problem-solving approach involves establishing an analysis based on convincing results, identifying potential solutions to be tested, deploying a concrete action plan, and monitoring the effects by evaluating the repercussions and adjusting intervention solutions as needed. The research team will collaborate with teams from the Quality, Evaluation, Performance, and Ethics (QEPE) departments to ensure that problem-solving tools chosen are in line with the strategies already established by the institution and that the results can continue to be measured by pre-selected indicators. Beyond using tools, problem-solving based on continuous improvement involves changing paradigms of thought and action. It also involves a change in the type of leadership and governance deployed to render increasingly more shared-decision making.

## The Integral Role of Patients as Partners

The program’s steps outlined above will be implemented and managed within the patient as partners approach. Patients as partners are individuals who have previously been treated for cancer and share their experiential knowledge about the disease and their experience of using the healthcare system to benefit other patients undergoing treatment (Pomey et al., 2015). Trained and mentored patients as partners are considered full team members who are a source of hope for patients and a source of recognition for providers (Usher, 2015; Bombard et al., 2018). As an aspect of the research, patients as partners, who are part of the research team, will be involved in each implemented mechanism (patient-centered meetings, talking spaces, and the agile problem-solving approach).

## The Proposed Integrative Organizational Model

This study seeks to demonstrate that multidisciplinary cancer care teams’ resilience derives from a repositioning of the relational dynamics within teams and the addition of both psychological and structural support resources. It has been estimated that nearly 60% of workplace teams that have encountered adverse (or unanticipated) events fail to return to their initial state, finding themselves trapped in a downward spiral affecting their confidence and performance (Hackman, 2002). For this reason, our participatory interventional approach is based on the theory of resource conservation (Hobfoll, 1989), suggesting that resources act at different levels (e.g., organizational, team, and individual) instrumentally as a pool of available resources allowing the individual and team to confront unforeseen events and to mitigate institutional dysfunctions. It is from this perspective that the concept of team resilience takes on its fuller meaning.

Team resilience presupposes the accumulation of and access to adequate structural and psychological resources that enable learning and improvement of collective and individual reactions following a major adverse event or a continual accumulation of disruptive events (Sutcliffe and Vogus, 2003). The proposed integrative organizational model shown in Figure 1 is based on this process perspective of resilience and emphasizes different levels of structural resources related to resilience (i.e., team and individual; Horne and Orr, 1998; Chênevert et al., 2020). These levels are interrelated and render it possible to create healthier interactional dynamics and enhanced conceptualizations of workplace adversities through the lens of team resilience (Gucciardi et al., 2018). This study posits that access to improved team resilience (Smith et al., 2018) will lead to improved team mental health, which, in turn, will lead to favorable organizational outcomes (Gittell et al., 2008; Tremblay et al., 2010). Therefore, this study’s integrative organizational model seeks to demonstrate that the deployment of the intervention to influence cancer care teams’ mental health status can only be effective to the extent that it promotes the emergence of psychological resources necessary for team resilience. Since the model is recursive, positive results will support team members’ mental health and access to the psychological resources for team resilience (Tremblay and Simard, 2005).

## Study Aims

The above considerations led us to propose the following research question: What is the role of team resilience in the link between the implementation of a participatory managerial approach, psychological resources, occupational health and their consequences on organizational outcomes? Two main objectives and related hypotheses include:

1. To evaluate associations between participatory components and team resilience. Therefore, this study aims to verify whether the implementation of each of the recommended intervention steps increases individual and team resilience among participating teams compared to team members who do not take part in the intervention.

*Hypothesis* 1: The implementation of the intervention will be significantly and positively related to psychological resources of resilience. Specifically, cancer care team members who take part in the intervention will report a significantly higher level of psychological resources of resilience relative to cancer care team members who do not take part in the intervention.

*Hypothesis* 2: Psychological resources of resilience will be significantly related to the team’s resilience. Specifically, participants who indicate a higher level of psychological resources of resilience will also indicate a higher level of team resilience.

*Hypothesis* 3: Psychological resources of resilience and team resilience will be significantly related to the team’s mental health status variables of burnout, engagement, quality of work life, and wellbeing. Specifically, participants and teams who indicate higher resilience will also indicate lower burnout and higher wellbeing.

2. To evaluate the link between team members’ improved mental health and organizational outcomes such as absenteeism, attrition, and organizational citizenship behavior.

*Hypothesis* 4: Team mental health status will be significantly and negatively related to absenteeism and attrition as well as positively related to organizational citizenship behavior.

## Materials and Methods

### Study Design and Procedures

This study was granted ethics approval by HEC Montreal, whereby all participants will be required to provide informed consent. The study is based on a multiple case study (i.e., qualitative and quantitative; Yin, 2014), including observations, semi-structured interviews, focus groups, and self-report questionnaires. This approach refers to the study of a particular phenomenon that can be linked to events and activities. The case study approach is a preferred approach for describing, exploring, and understanding a phenomenon in its “real context” (Yin, 2014). For this study, the phenomenon under investigation is the implementation of a participatory interventional approach aimed at fostering team resilience. The cases will include the five cancer teams where the intervention will be deployed. The study population includes approximately 600 healthcare professionals associated with 20 cancer teams (an average of roughly 30 members per team) from three institutions in two regions of Quebec. The proposed intervention will involve five of these teams for an approximate total of 150 healthcare professionals. The team members who will not participate in the intervention will act as a control group.

Given our close affiliation with the participating teams and their formal commitment, we expect response rates between 60% and 70% (Reimer et al., 2017), which will support the statistical models used. The multidisciplinary cancer teams include (but are not limited to) chemotherapy nurses, nurse navigators, nutritionists, occupational therapists, oncologists, pathologists, patient care workers, pharmacists, physiotherapists, psychologists, social workers, and surgeons. Of note, careful attention will be paid to ensure a balanced composition of participating teams consisting of equivalent categories of healthcare professionals to minimize the extraneous effect of team composition on study outcomes. This study will also include patients as partners as team members. The participating teams will be involved in treating various types of cancers and will be selected by health-services management, together with the department heads and the teams themselves, who will need to be mobilized and volunteer. This study prioritizes teams that are not subject to “contamination,” i.e., with as few professionals as possible moving between teams.

The study will use various measures to assess the impact of the implemented intervention. To this end, three measurement periods are planned: before the deployment of the participatory intervention (T0) and after the deployment of the components of the participatory intervention (T1 = T0 + 12 months). A third measurement phase (T2 = T0 + 24 months) will be planned once the participatory intervention is well established within the teams. Table 2 shows the three study measurement phases. To evaluate whether the results observed are, indeed, related to the intervention and not a consequence of exogenous factors, the members of the other cancer teams of the three participating establishments will be subjected to the same questionnaires as the members of the five teams (total of 15 teams: 20-5). Specifically, they will act as a control group and will be surveyed using the same measurement tools at all three stages.

**Table 2:** Study measurement steps.

Steps	Description	Anticipated sample size (*N*)	Data collected
1	Pre-deployment	375	Online self-report questionnaires Observations Focus group
2	Post-deployment (12 months)	236	Online self-report questionnaires Semi-structured interviews Focus group
3	Post-deployment (24 months)	149	Online self-report questionnaire Semi-structured interviews Focus group

In previous studies (under similar contexts; Preacher and Hayes, 2008), the response rate varies between 60% and 70%, depending on whether respondents participate in the implementation. Therefore, we can anticipate a sample of approximately *N* = 375 for T0 (i.e., 270 for non-participants—60% of 450—and 105 for participants—70% of 150), *N* = 236 for T1 (i.e., 162 for non-participants 60% of 270—and 74 for participants—70% of 105) and *N* = 149 for T2 (i.e., 97 for non-participants—60% of 162—and 52 for participants—70% of 74). Regarding the margin of error of sampling, the calculation of the error interval indicates 3.10% for T0, 3.89% for T1 and 4.88% for T2. The objectives of the three measurement times are to reduce the limitations of previous studies faced with the problem of common variance, which artificially increases the link between variables (Podsakoff and Organ, 1986), and to establish a causal link between the study variables, thus respecting the temporal sequence of the proposed integrative organizational model. Additionally, objective variables such as the number of absences and attrition/staff turnover will reduce the bias of self-reported measures.

### First Type of Data Collection: Quantitative

An online self-report questionnaire designed for this study using validated instruments, including 13 measurement tools (i.e., support, recognition, empowerment, organizational justice, individual resilience, psychological safety, work climate, team resilience, workplace burnout, engagement, quality of work life, wellbeing, and organizational citizenship behavior), will be sent directly to participants in French or English, depending on the language of correspondence desired. The measurement tools used have all been previously validated in French and English with healthcare personnel.

### Measures

#### Sociodemographic Characteristics

An author-generated self-report questionnaire will be used to gather information on participants’ age, sex, profession, work status, child dependents, work schedule, job and hospital seniority, work experience, and supervision responsibilities.

#### Psychological Resources for Resilience

Seven dimensions of the psychological resources of resilience will be measured. Support will be measured using the three items of the scale by Eisenberge et al. (1986). Example items include “I know that I can count on my supervisor if I have a problem,” with internal consistency ranging from 0.74 to 0.95 (Eisenberge et al., 1986). Recognition will be measured using the six items of the scale by Jourdain and Chênevert (2020). Example items include “My direct supervisor congratulates me often for my efforts,” with internal consistency ranging from 0.81 to 0.90 (Jourdain and Chênevert, 2020). Empowerment will be measured using the 12 items of the scale by Spreitzer (1995). Example items include “I have enough power to accomplish my tasks efficiently,” with internal consistency ranging from 0.76 to 0.88 (Spreitzer, 1995). Organizational justice will be measured using the six items of the scale by Niehoff and Moorman (1993). Example items include “The managers make sure that all employees’ concerns are heard before making decisions,” with internal consistency ranging from 0.74 to 0.92 (Niehoff and Moorman, 1993). Individual resilience will be measured using the 10 items of the Connor-Davidson Resilience Scale (CD-RISC; Campbell-Sills and Stein, 2007). Example items include, “I do not easily become discouraged after a failure,” with internal consistency ranging from 0.87 to 0.96 (Campbell-Sills and Stein, 2007). Psychological safety will be measured using the seven items of the brief scale by Edmondson (1999). Example items include, “If you make a mistake on this team, it is often held against you,” with internal consistency ranging from 0.82 to 0.96 (Edmondson, 1999). Work climate will be measured using the scale of Barki and Hartwick (2003). Example items include “Individuals often place obstacles in each other’s way,” with internal consistency ranging from 0.75 to 0.89 (Barki and Hartwick, 2003).

#### Team Resilience

Team resilience will be measured using the seven items of the measurement tool by Mallak (1998). Example items include, “In difficult situations, my team tries to see the positive side of things,” with internal consistency ranging from 0.78 and 0.91.

#### Team Mental Health Status

Four dimensions of mental health status will be considered. Burnout will be measured using the two items of the brief scale provided by Maslach and Jackson, validated by West et al. (2012). Example items include, “I feel emotionally drained by my work” (West et al., 2012). Engagement will be measured using the nine items of the scale provided by Schaufeli et al. (2006). Example items include, “I’m full of energy for my work,” with internal consistency ranging from 0.80 to 0.90 (Schaufeli et al., 2006). Wellbeing will be measured using the five items of the WHO measurement tool (Psychiatric Research Unit, 1999). Example items include, “In the last 2 weeks, I have felt good and in a good mood,” with internal consistency ranging from 0.74 and 0.87 (Psychiatric Research Unit, 1999). Quality of life at work will be measured using the 16-item scale designed by Delmas et al. (2001). Example items include, “To what extent does your job give you the opportunity to be successful in expressing the uniqueness of your personality,” with internal consistency ranging from 0.76 to 0.88 (Delmas et al., 2001).

#### Organizational Outcomes

Three dimensions of organizational outcomes will be considered. Two organizational outcomes will be measured using the number and frequency of days of absence obtained from employee files and attrition/staff voluntary turnover. Organizational citizenship behavior will be measured using the 10 items of the scale by Organ (1988). Example items include, “Offered suggestions for improving the work environment,” with internal consistency ranging from 0.87 to 0.96 (Organ, 1988). Specific individual characteristics will be controlled for in the statistical model, including age, sex, marital status, number of dependents, occupation, seniority in the current position and within the institution, supervisory responsibilities, work experience, and work schedule.

### Statistical Analysis

Descriptive analysis will serve to assess personal and team resilience resources, overall mental health status of teams, and organizational outcomes. Analyses of differences in means (e.g., *t*-test and cross-tabulation) will be used to estimate potential differences between all study variables. The proposed integrative organizational model (Figure 1) will be empirically tested using structural equation modeling. Table 3 shows questionnaires corresponding to each data collection phase of the study. The first phase of model development will be carried out using the sample (*N* = 375). Following the approach suggested by Anderson and Gerbing (1988), the study will first verify the measurement model’s goodness-of-fit using confirmatory factor analysis and then estimate the structural model. In a second “confirmatory” phase, the study will test the final structural model obtained in phase 1 (Anderson and Gerbing, 1988; Bagozzi and Yi, 1988) and verify our hypotheses.

**Table 3:** Quantitative date collection per study measurement steps.

Measurement steps	Constructs	Questionnaires	Number of items
T0	Demographic information	Author generated	10
T0-T1-T2	Psychological resources for resilience	Support (Eisenberge et al., 1986) Recognition (Jourdain and Chênevert, 2020) Empowerment (Spreitzer, 1995) Organizational justice (Niehoff and Moorman, 1993) Individual resilience (Campbell-Sills and Stein, 2007) Psychological safety (Edmondson, 1999) Work climate (Barki and Hartwick, 2003)	3 6 12 6 10 7 5
T0-T1-T2	Team resilience	Team resilience (Mallak, 1998)	10
T0-T1-T2	Team mental health status	Professional burnout (West et al., 2012) Engagement (Schaufeli et al., 2006) Wellbeing (Psychiatric Research Unit, 1999) Quality of work life (Delmas et al., 2001)	2 9 5 16
T0-T1-T2	Organizational outcomes	Turn over^*^ Absenteeism^*^ Organizational citizenship behavior (Organ, 1988)	N/A N/A 10

* *Data obtained via human resources records*.

The goodness-of-fit of the structural equation model will be assessed based on several indices. Thus, a Root Mean Square Error of Approximation (RMSEA) of less than 0.05 indicates a reasonable degree of fit, and values up to 0.08 indicate a reasonable error of approximation in the population (Byrne, 1998). A Normed Fit Index (NFI) and a Comparative Fit Index (CFI) between 0.90 and 1 also indicate the presence of a well-fitted model (Bentler, 1992; Hu and Bentler, 1995). We will also report the classical chi-square statistic (*χ*^2^) to compare the fit quality of nested models. As recommended by Preacher and Hayes (2008), we will estimate the significance of indirect effects using the Bootstrap method (Sobel, 1982; Shrout and Bolger, 2002; Preacher and Hayes, 2008). These statistical analysis models will make it possible to identify the weight of each form of resilience in order to estimate which are the most critical determinants of the team’s mental health status. They will also be used to evaluate the mediating role of the team’s mental health status on the potential link between resilience and organizational outcomes.

### Second Type of Data Collection: Qualitative

First, in (T0), qualitative data will provide an initial portrait of the teams regarding climate and resources related to resilience. The observation of multidisciplinary activities within each team will be carried out over 4 weeks (60 h/case; Bilodeau et al., 2015). The observation will make it possible to document teams’ functioning and structural resources (e.g., meetings, conflict management, and leadership). Semi-structured interviews with professionals, including patients as partners and support staff, lasting approximately 60 min will be conducted (*n* = 10/case). The interview guide will focus on perceptions of the psychological resources of resilience (e.g., support, recognition, and work climate). A focus group (*n* = 1/case) will bring together 6–10 professionals and patients (Krueger and Casey, 2009). The discussion will concentrate on functioning, strengths, and areas for improvement. A purposive sampling strategy (Guest et al., 2013) will be used to target a variety of healthcare professionals (e.g., physicians, nurses, nutritionists, and pharmacists).

The use of different qualitative approaches will increase richness of findings, while identifying individual and contextual circumstances of the complex environment in which healthcare professionals work (Lambert and Loiselle, 2008). The transcribed data from observations, semi-structured interviews, and focus groups will be subjected to an iterative content analysis that includes the following activities: condensation, data presentation, and development and verification of conclusions (Miles et al., 2014). A codebook will be constructed from the integrative organizational model of the study and enhanced during the analysis. The data will be triangulated to validate the findings. Subsequently, in (T2), the data collected will make it possible to document and better understand potential links between the components of the participatory interventional approach and resilience. Observations, semi-structured interviews, and a focus group will again be carried out according to the procedure explained above.

It should be noted that the semi-structured interviews will explore the participants’ experience with activities related to the participatory interventional approach and associated perceived benefits and challenges. The focus group will explore participants’ perceptions of team resilience and the effectiveness of the intervention. The results of each case will be pooled. The cases will then be contrasted to describe their particularities. The following criteria will ensure data quality: internal credibility/validity (triangulation of data, cross-jurisdictional validation), reliability (validation of specific data by participants), procedural accountability (documentation of the research process), and external transferability/validity (detailed description of the context; Miles et al., 2014).

## Discussion

This proposed study responds to concerns expressed by various oncology stakeholders, particularly in terms of reducing distress and burnout and improving the resilience and working climate of multidisciplinary teams. We anticipate several outcomes associated with this study. First, we expect that the intervention will reduce absenteeism and attrition/staff voluntary turnover, which, in turn, will reduce the overload of team members who regularly have to cope with staff shortages. Second, we expect to develop a better understanding of how to refine the study intervention, in part, through knowledge gained *via* post-intervention deployment interviews. Third, we hope to validate the proposed integrative organizational model (Figure 1) and, consequently, enhance scientific knowledge concerning the complementary roles of individual and collective resilience on team mental health status among multidisciplinary team members. Data collection across several measurement phases may shed further insights into resilience and the withdrawal mechanisms of absenteeism and attrition/staff turnover. The study results will also provide a better understanding of the potential mediating role of team members’ mental health status on the relationship between resilience and organizational outcomes. Understanding these processes is fundamental to future research in the area of team resilience. Fourth, we expect to empirically support the participatory interventional approach, thus, moving beyond previous burnout and wellbeing frameworks. To this end, all tools developed for and implemented in this study (e.g., patient-centered meetings) will serve as potential benefits for future research and organizational management. Fifth, we expect that our interventional approach, if corroborated further, will also be adopted by additional multidisciplinary teams (or public safety personnel) facing workplace adversity and operational stress, e.g., firefighters, military, paramedics, police, other healthcare teams. Last, following Traylor et al. (2021), we anticipate that our findings will provide multidisciplinary teams with knowledge concerning how to maintain their effectiveness, especially in the context of extreme environments or crises.

Many healthcare institutions have shown an interest in implementing the participatory interventional approach proposed herein and benefiting from its derivatives. Moreover, this study is part of a broader knowledge development program, as a first demonstration study is already underway at the Centre Intégré Universitaire de Santé et de Services Sociaux (CIUSSS) Centre Ouest (specifically the Jewish General Hospital) in Montreal, Quebec. The validation of the participatory interventional approach across five different cancer care teams will enable the research team associated with this study to increase transferability. If the participatory interventional approach is positively validated, the research team intends to apply the approach to a larger number of teams and develop formal training materials to ensure the transfer of skills and tools in a *training of trainers* approach. Ultimately, in collaboration with various knowledge users, the research team intends to develop multidisciplinary team certification.

## Ethics Statement

The studies involving human participants were reviewed and approved by the Research Ethics Board (REB) of HEC Montreal. Written informed consent was not provided because participant recruitment and data collection have yet to commence.

## Author Contributions

DC and CL co-developed the project. DC developed the conceptual model. DC and TB prepared the overall structure of the manuscript. All authors contributed to the article and approved the submitted version.

## Funding

The study described in this manuscript was funded by the Social Sciences and Humanities Research Council of Canada (435-2020-1370). This funding agency had no role in the design of the study or the collection, analysis, and interpretation of data or in writing the manuscript, apart from their financial contribution.

## Conflict of Interest

The authors declare that the research was conducted in the absence of any commercial or financial relationships that could be construed as a potential conflict of interest.

## Publisher’s Note

All claims expressed in this article are solely those of the authors and do not necessarily represent those of their affiliated organizations, or those of the publisher, the editors and the reviewers. Any product that may be evaluated in this article, or claim that may be made by its manufacturer, is not guaranteed or endorsed by the publisher.

## Abbreviations

CFI: Comparative fit index
CD-RISC: Connor-Davidson resilience scale
NFI: Normed fit index
RMSEA: Root mean square error of approximation
QEPE: Quality, evaluation, performance, and ethics
